# Analysis of alternative splicing events for cancer diagnosis using a multiplexing nanophotonic biosensor

**DOI:** 10.1038/srep41368

**Published:** 2017-01-25

**Authors:** César S. Huertas, Santos Domínguez-Zotes, Laura M. Lechuga

**Affiliations:** 1 Nanobiosensors and Bioanalytical Applications Group, Catalan Institute of Nanoscience and Nanotechnology (ICN2), CSIC, The Barcelona Institute of Science and Technology, and CIBER-BBN, Campus UAB, Bellaterra, 08193 Barcelona, Spain

## Abstract

Personalized medicine is a promising tool not only for prevention, screening and development of more efficient treatment strategies, but also for diminishing the side effects caused by current therapies. Deciphering gene regulation pathways provides a reliable prognostic analysis to elucidate the origin of grave diseases and facilitate the selection of the most adequate treatment for each individual. Alternative splicing of mRNA precursors is one of these gene regulation pathways and enables cells to generate different protein outputs from the same gene depending on their developmental or homeostatic status. Its deregulation is strongly linked to disease onset and progression constituting a relevant and innovative class of biomarker. Herein we report a highly selective and sensitive nanophotonic biosensor based on the direct monitoring of the aberrant alternative splicing of *Fas* gene. Unlike conventional methods, the nanobiosensor performs a real-time detection of the specific isoforms in the fM-pM range without any cDNA synthesis or PCR amplification requirements. The nanobiosensor has been proven isoform-specific with no crosshybridization, greatly minimizing detection biases. The demonstrated high sensitivity and specificity make our nanobiosensor ideal for examining significant tumor-associated expression shifts of alternatively spliced isoforms for the early and accurate theranostics of cancer.

Gene expression is controlled by a complex regulatory network highly structured where diverse mechanisms act at different expression levels, aiding the cells to adapt successfully to environmental changes^1^. However, the alteration of gene expression may influence the fate of the cells and its consequences can be devastating, resulting in the origin of grave diseases^2^. These mechanisms are considered as a brand-new class of biomarkers for the diagnosis of many malignancies, including cancer, providing more informative, specific and accurate analyses. In addition, because of their dynamic nature and their potential reversibility, they constitute appealing therapeutic targets for cancer treatment. The current progress in nanotechnology together with the increasing knowledge in epigenetics have promoted the development of biosensor devices which stand out among other analytical techniques due to their capabilities for fast, sensitive and precise evaluation of such mechanisms, holding enormous potential as technological solutions for rapid and reliable biomedical analysis^3^,^4^,^5^,^6^.

Alternative splicing is one of the above mentioned mechanisms, acting at the post-transcriptional level by the alternative edition of a pre-mRNA in order to give rise to different mRNA templates^7^. Each template will produce proteins with different functions, which in some cases can present opposed effects. Most cancers are associated with a switch in the splicing pattern of specific isoforms that provides cancer cells with proliferative capacity and survival properties^8^. Thus, the early detection of aberrant switches in the splicing pattern will permit the prognosis of the cancer onset as well as specific clues for the reversion of the tumorigenic process^9^.

Existing analytical techniques for the specific detection of splicing isoforms are highly complex for clinical purposes as they normally involve pre-amplification steps and often exhibit complications in discerning between closely related isoforms due to cross-hybridization^10^,^11^. Recently, we have developed a biosensor methodology using a standard Surface Plasmon Resonance (SPR) sensor^5^ for the specific detection of mRNA isoforms generated by alternative splicing and demonstrated its feasibility by the direct detection of *Fas* gene isoforms in model HeLa cell lineages in a straightforward and fast analysis. However, although the methodology was in good agreement with the gold-standard technology (qRT-PCR), the SPR sensitivity, which is in the picomolar-nanomolar (pM-nM) scale (387 pM LOD), may compromise its further use in clinical diagnosis. The levels of mRNA isoforms in normal and cancer tissues can be found in the low pM concentration^12^, requiring a more sensitive detection.

Here we report a novel biosensor based on bimodal nanowaveguide interferometers (BiMW biosensor) for the highly sensitive and selective analysis of alternatively spliced mRNA isoforms. The BiMW biosensor^13^ consists of sixteen interferometric waveguides of silicon nitride (Si_3_N_4_) fabricated over a silicon substrate via standard microelectronic technology (Fig. 1A), which is further integrated in a Lab-on-Chip platform^14^. This label-free optical nanosensor is capable of detecting a direct DNA/RNA sequence hybridization by the evanescent field interaction of two different modes of the light that propagate along the nanowaveguides. The final interferometric signal is then modulated, generating an easily-readable linear signal^15^. Our novel device has been described as one of the most sensitive optical interferometric sensors^13^ and its applicability for direct small RNA detection has been recently demonstrated by the ultrasensitive detection of micro-RNAs at the attomolar level^16^ but it has never been applied for long mRNA sequences. The proposed biosensor involves the specific detection of *Fas* gene isoforms, i.e. *Fas*567 and *Fas*57, generated by alternative splicing of its pre-mRNA, using independent interferometers on the same chip (Fig. 1B). While *Fas*567 isoform encodes for an apoptotic receptor, activating the characteristic signalling cascade of the extrinsic apoptotic pathway, *Fas*57 leads to a soluble protein with an anti-apoptotic function. *Fas*57 isoform is overexpressed in cancer cells and contributes to cancer aggressiveness^17^. Thus, the expression ratio of mRNA *Fas* isoforms represents a potential biomarker for the early diagnosis of cancer^8^. Here we demonstrate the high sensitivity achieved (fM-pM levels) by the nanobiosensor in a rapid (less than 30 min) and label-free detection of splicing variants without either cDNA conversion or PCR amplification, corroborating the potential of this innovative device for the fast screening of alternative splicing events as biomarkers for cancer prognosis.

## Results

### Optimization of the biofunctionalization procedure

**Figure 2:**
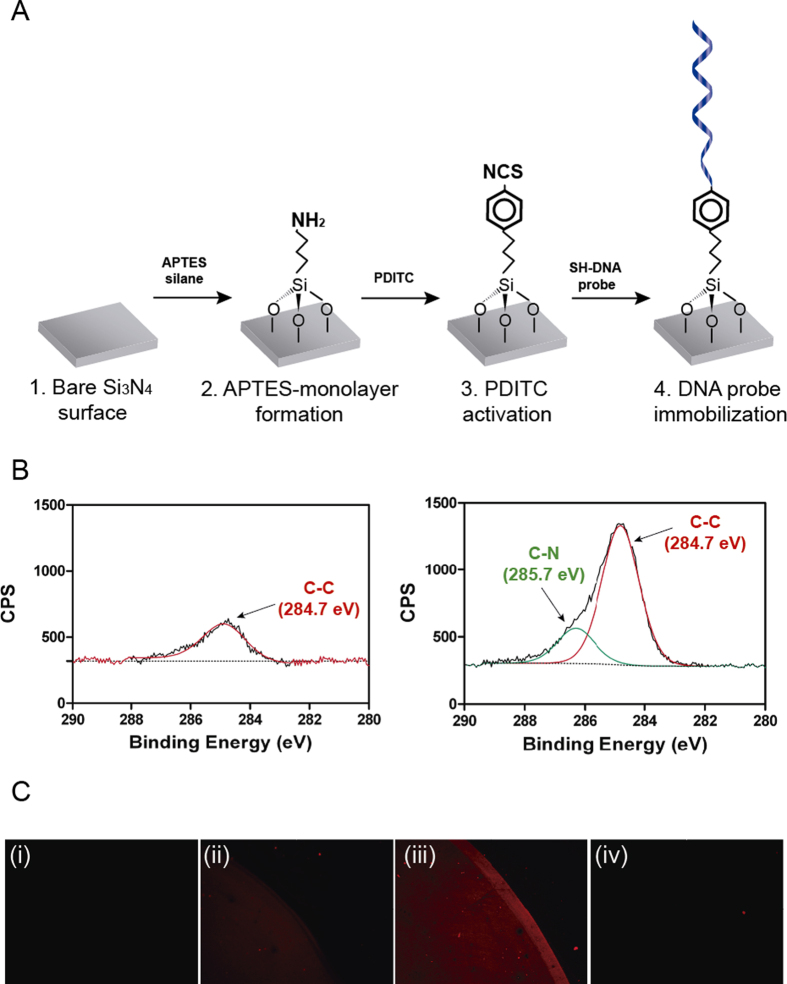
Characterization of the biofunctionalization process on Si_3_N_4_ surfaces. (**A**) Scheme of the protocol followed for Si_3_N_4_ sensor surface biofunctionalization. (**B**) XPS analysis of the binding energy for C1s bonds in untreated (*left*) and APTES-modified Si_3_N_4_ surfaces (*right*). (**C**) Fluorescent signals from the Cy3-*Fas*57 target for (i) untreated, (ii) PDITC-activated, (iii) complementary DNA-functionalized, and (iv) non-complementary DNA-functionalized sensor surfaces.

First, we focused on the optimization of the biofunctionalization conditions to be employed in the sensor surface, a key step in the development of a biosensor. One of the most widespread used strategies for silicon-based surfaces is the functionalization with the 3-aminopropyltriethoxy silane (APTES) as it generates a silane monolayer on the sensor surface under controlled conditions. This silane provides silicon surfaces with functional amino (NH_2_) groups^18^,^19^ that can incorporate different crosslinkers for the immobilization of biomolecules carrying specific linker groups. Figure 2A shows the strategy followed for the functionalization of the silicon nitride (Si_3_N_4_) BiMW biosensor surface. Briefly, we obtained a homogeneous monolayer of APTES silane under controlled conditions of humidity, O_2_ content and pressure. This monolayer was activated with the p-Phenylene diisothiocyanate (PDITC) crosslinker, a small homobifunctional crosslinker which is commonly used in bioconjugation chemistry^20^ and permit the formation of versatile monolayers by the linkage of different molecules. In the final step, the PDITC was finally linked covalently with a specific DNA probe.

The silanization process was verified by X-ray Photoelectron Spectroscopy (XPS). We performed a narrow scan of the C1s signal to examine the chemical states of carbon on the APTES-modified and untreated surfaces. The deconvolution of the C1s peak for the modified surface with APTES showed a main band at 284.7 eV, due to the elongation of the hydrocarbon chain in contrast with the soft peak from the untreated surface, probably due to organic contamination (Fig. 2B). More importantly, a new peak contribution was detected at 285.7 eV, attributable to C-N bonds. These results corroborated the successful grafting of the silane onto the surface and evidenced the formation of a silane monolayer onto the Si_3_N_4_ surface with terminal amine functional groups for further attachment of biomolecules. All electron binding energies carbon peak positions were derived from the literature for other similar systems^21^,^22^.

Next, we studied the covalent binding of DNA probes to the APTES monolayer through the PDITC crosslinker^20^. Isothiocyanate (R-NCS) group is very stable in solution and it is known to be reactive either to primary amine groups, yielding thiourea bonds, or to thiol groups, forming thiocarbamate bonds. Conjugation of biomolecules to NCS groups proceeds mostly through reaction with thiol groups in a fast reaction^23^. Taking advantage of this dual reactivity, we established a protocol for the modification of aminated surfaces with PDITC and subsequent coupling to the thiol-modified DNA probes through thiocarbamate bonds. Thus, the APTES-modified surfaces were reacted with PDITC. Activated surfaces where incubated with the SH-*Fas*57 probe (Table 1) in Na_2_CO_3_ buffer at pH 9.5. Basic pH leads to the reactive state of the APTES primary amine, promoting the reaction with the NCS group. To assess not only the proper bioconjugation but also the applicability of the biofunctionalization for target capture, the hybridization of the SH-*Fas*57 probe was characterized through fluorescence assays. Biofunctionalized surfaces were incubated with a solution containing 3′-Cyanine 3 (Cy3) modified DNA sequences complementary to the immobilized probe (Cy3-*Fas*57; Table 1) in 5× SSC buffer (pH 7). Untreated surfaces and PDITC-activated surfaces did not show any fluorescence for the Cy3-*Fas*57 sequence, indicating that none unspecific physical adsorption took place onto the surface (Fig. 2C(i) and (ii)). A clearly specific signal for the surface functionalized with the SH-*Fas*57 probe (Fig. 2C(iii)) was appreciated. In addition, no fluorescent emission was detected in a second surface with a non-complementary probe (SH-*BCL-X*_*L*_ probe, Table 1) (Fig. 2C(iv)), confirming the specificity of the methodology. Such results also agreed with the evaluation of static contact angle (Table 2), which showed increasing hydrophilicity after PDITC activation and DNA functionalization, 59.6° and 51.7° respectively, in contrast with the APTES modified surfaces (67.1°). The contact angles values for untreated and APTES- modified surfaces are in good correlation with other published results^24^.

### BiMW biosensor biofunctionalization

**Figure 3:**
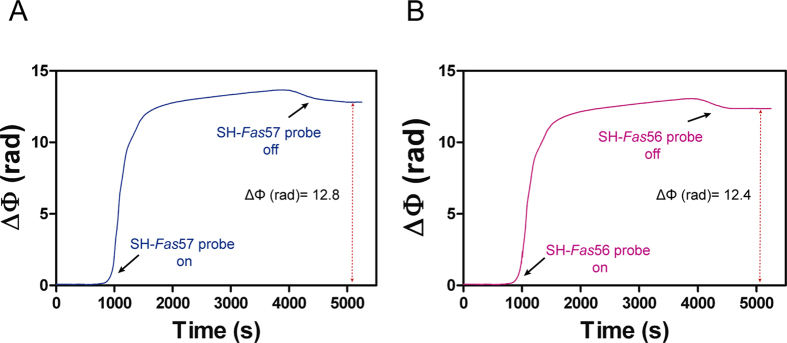
Biofunctionalization process carried out on the BiMW surface. Real-time immobilization signals of (**A**) SH-*Fas*57 probe and (**B**) SH-*Fas*56 probe. Black arrows indicate the entrance (‘on’) and exit (‘off’) of the DNA probes in the sensor area during the interaction with the sensor surface.

Once assessed the proper conditions for the sensor biofunctionalization, we evaluated the analytical performance of the BiMW device for the direct detection of *Fas* gene isoforms. We functionalized the BiMW sensor surface following the optimized protocol. In order to independently analyze each isoform, the different probes employed were *in-situ* immobilized separately in two different sensor channels after the PDITC-modification. Thus, the BiMW sensor chip was placed into the microfluidic cell and two different microfluidic channels were supplied with a solution containing either SH-*Fas*57 or SH-*Fas*56 probes and 6-Mercapto-1-hexanol (MCH) lateral spacer to improve the target accessibility by minimizing steric hindrance forces. They were mixed at a 20:1 molar ratio as reported in a previous work^5^. The different DNA probes employed consist of the particular exon-junction sequence of each isoform. Splice-junctions (i.e. complementary to exon sequences flanking the splice junction) represent a unique sequence feature of each transcript, which is essential in order to obtain the specific detection of each mRNA isoform. The covalent binding of the SH-DNA probes to the PDITC-activated surface was then monitored in real-time by the analysis of the modulated interferometric signals of the BiMW sensor (Fig. 3). The sensor gave an increment in the response (ΔΦ (rad)) of 12.8 and 12.4 for SH-*Fas*57 and SH-*Fas*56, respectively, which corroborated the formation of the monolayer in-flow and demonstrated the applicability of the optimized biofunctionalization protocol for DNA functionalization of Si_3_N_4_ surfaces.

### Alternative splicing BiMW biosensor assessment

For the biosensor demonstration, we employed DNA homologous sequences of 200-nucleotide length containing the exon-junction sequences of both *Fas* gene isoforms (Table 1) as a model^5^. Two key factors are required for the direct detection of *Fas* isoforms: the selectivity and sensitivity. The former is necessary due to the similarity between the isoform sequences, which complicates their specific detection with no crosshybridization of the off-targets. The latter is important because of the low concentration of such isoforms found in cells (low-pM concentrations). To assess the selectivity, we monitored the biosensor response to the hybridization with a concentration of 50 pM of each isoform with the different probes. For detection assays, we employed the hybridization conditions previously established with the SPR biosensor (i.e. hybridization buffer: 3× SSC/FA45%), specifically optimized to reduce the non-specific signals^5^. Hybridization solutions containing an adequate percentage of formamide reduce the presence of secondary structures and increase the specificity by lowering the melting temperature of oligonucleotides^25^. The highly stringent conditions of our hybridization buffer, mainly provided by the formamide content, provoke a drastic effect on specificity and are thoroughly optimized to favor the detection of the isoforms with their specific probes compared to the alternative ones. These conditions of specificity operate very efficiently in a range of 40–63% of GC content in the probe sequence^5^, which is the common percentage range employed for DNA probes, highlighting its potential for the analysis of alternative splicing events of different genes and contexts.

**Figure 4:**
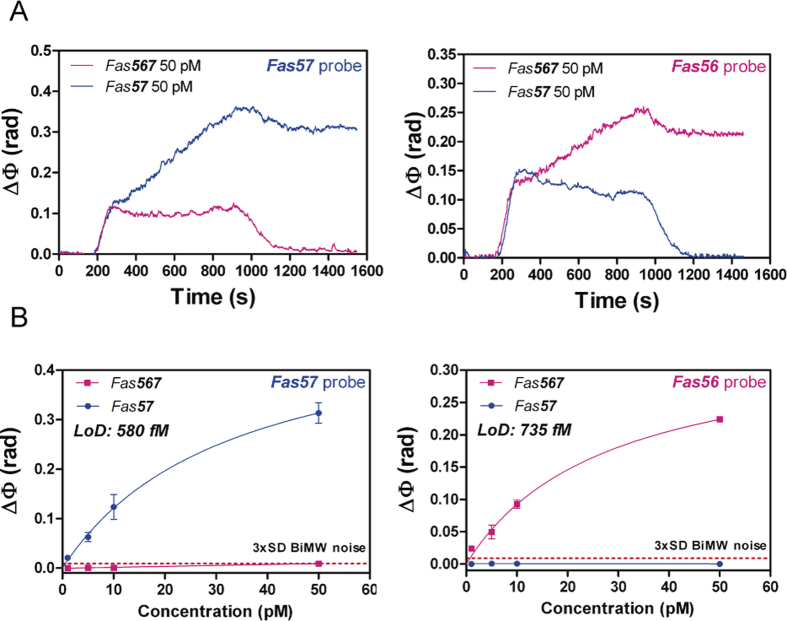
BiMW detection of Fas gene alternative splicing isoforms. (**A**) BiMW real-time sensograms of *Fas* isoforms for *Fas*57 probe (*left*) and *Fas*56 probe (*right*). (**B**) BiMW calibration curves for *Fas*57 probe (*left*) and *Fas*56 probe (*right*). Solid lines *(blue* and *pink*) correspond to the non-linear fit of the calibration curves. Red dashed line corresponds to 3xSD BiMW noise, which is the limit for the minimum signal detectable (All data show mean ± SD of triplicate measurements).

As can be appreciated in Fig. 4A, the real-time monitored signal of each isoform led to an increase in the phase shift after the hybridization with the monolayer containing their specific probes. The signal corresponding to the off-targets returned to the former baseline levels, which indicated that no hybridization took place. These results demonstrated the remarkable specificity of the methodology and the success in isoform discrimination by the BiMW biosensor, crucial to provide reliable evaluation of the isoform ratio for a precise diagnosis. Moreover, the employed concentration of 50 pM led to a clearly detectable sensor response within less than 30 minutes, which evidenced the high sensitivity of the BiMW biosensor for the fast analysis of very low target concentration.

Having demonstrated the specificity, the next step was to further demonstrate the sensitivity level of the novel BiMW biosensor. For that, we performed a calibration curve for each isoform. We employed different concentrations of *Fas*57 and *Fas*567 from standard solutions, ranging from 1 pM to 50 pM and fitted the signals to a non-linear curve. As can be observed in Fig. 4B, the BiMW device was able to precisely discriminate the non-complementary isoforms and clearly detect the specific ones at all the measured concentrations. The LODs obtained were of 580 and 735 fM for *Fas*57 (R^2^ = 0.98) and *Fas*567 (R^2^ = 0.98) probes, respectively. The BiMW biosensor performance reflected an improvement of three orders of magnitude compared with the one achieved with the SPR sensor (387 and 438 pM for each isoform). The calibration curves covered concentrations within the fM-pM range, which is in the physiological concentration range of endogenous mRNA sequences, allowing their reliable and direct detection without the need for any amplification process.

## Discussion

In conclusion, we have demonstrated the highly specific and sensitive detection of mRNA isoforms for the monitoring of deregulated splicing events for diagnostic purposes. The BiMW biosensor allowed for the parallel detection of both isoforms at concentrations as low as 580 fM, improving in three orders of magnitude the one achieved by SPR biosensor and representing, as far as we are concerned, the most sensitive approach for the analysis of alternatively spliced isoforms in a PCR amplification-free detection. The methodology showed a 100% discrimination of the off-targets, constituting an excellent tool for alternative splicing event monitoring in any tissue or biofluid, where the isoform concentrations are found in very low amounts. The biosensor strategy we propose here can be easily adapted for the study of the alternative splicing events occurring in a wide variety of genes by simply modifying the design of the DNA probes. However, it is worth mentioning that, in order to analyze real samples and ensure the standardization of our methodology, a simple RNA fragmentation protocol^5^ must be performed to unify the isoforms’ length and match the one of the standard DNA homologous employed for the calibration. Further studies should be done related to the analysis of mRNA isoform switches in tissues in order to confirm its utility in isoform rate prediction. Moreover, it has been demonstrated that the presence of circulating mRNA in blood stream from cancer patients can be associated with the tumor progression^25^ which could be a promising approach for detecting deregulation of alternative splicing events in a less-invasive analysis. We believe that this new nanotechnology constitutes a step forward for the development of a multiplexing biosensor in order to build complete regulation panels capable of deciphering the different routes taken by the cell, from the DNA to the final protein, and elucidate which one is bringing forth the tumorigenic process.

## Methods

### BiMW biosensor

The BiMW biosensor has been previously developed in our Group^13^. The device is fabricated at wafer level using standard microelectronic technology in Clean Room facilities. An array of 16 interferometers of 30 mm length is integrated over a chip. A scheme of the BiMW biosensor working principle is shown in Figure S1. In brief, light from a polarized laser source (Helium-Neon, λ0 = 660 nm) is first confined into a nanometric height rib waveguide (4 μm width × 1.5 nm height) designed to support a single transversal mode (150 nm thickness) (Section A). After some distance, this fundamental mode is coupled into a vertically bimodal section (300–350 nm thickness) through a step junction, i.e. a modal splitter (Section B). At the step junction, due to the abrupt increase of the height of the waveguide core, it splits in two modes, the fundamental and the first order modes. These two modes propagate until the end of the device, resulting in an intensity distribution which depends on the phase difference between the two modes accumulated along the propagation length. A sensing window is opened along the bimodal section of the waveguide (Section C), where the evanescent field is in contact with the external medium and is sensitive to variations of the refractive index of the environment. Due to the different confinement factors of the propagating modes, the first order mode is the main responsible for the sensing of changes occurring on the waveguide surface while the fundamental mode can be considered as a virtual reference. The superposition of these two modes results in an intensity distribution giving rise to an interferometric signal whose variations can be related to the amount of stimulus variation and is recorded by a two-section photodetector at the end of the waveguide (Figure S1, Section D). Finally, a developed all-optical phase modulation method based on Fourier transform deconvolution^15^ is applied, modulating the interference signal to a real-time linear one, avoiding phase ambiguities.

### Reagents and buffer solutions

The list of reagents, buffers and solvents used in this work are provided in the Suplementary material.

### Polymeric flow cell fabrication

The flow cell employed contained Polydimethylsiloxane (PDMS) channels and is fabricated by polymer casting using a methacrylate mold. Elastomer and curing agent are mixed in a ratio 10:1 and air bubbles originating from their mixing are removed by a vacuum degas process. The mixture is cured for 1 hour at 75 °C to ensure cross-linking of the polymer. After this thermal process, the flow cell can be released from the methacrylate mold. The high hydrophobicity of the resulting PDMS channels is reduced by application of a Polyethylene glycol coating (PEG200, Sigma-Aldrich) after an ozone plasma treatment to expose functional groups on the polymer surface. Finally, PolyTetraFluoroEthylene (PTFE) tubes are inserted at each channel extremities and fixed with PDMS to avoid leakages or air insertion. The fluidic cell has four independent channels, with a volume of 15 μl each. Each fluidic channel addresses a group of four BiMW sensors on the fabricated chips.

### Si_3_N_4_-surface cleaning, activation and biofunctionalization

Si_3_N_4_ surfaces undergo an accurate cleaning prior to its use. First the chips are sonicated sequentially in acetone, ethanol and water, 2 min each. The cleaning process was finished by ultrasonication of the Si_3_N_4_ surfaces in a 1:1 metanol/hydrochloric acid (MetOH/HCl) solution to remove other inorganic contamination. The chips are rinsed with milli-Q H_2_O and dried under a N_2_ stream. After chip cleaning, a layer of active hydroxyl group was generated on the surface by UV/O_3_ activation (BioForce Nanosciences, USA) and exposition to 15% nitric acid solution, revealing the oxide groups on the Si_3_N_4_ surface. Clean and hydroxyl-activated chips were immediately transferred in a 1% v/v APTES solution prepared in water-free toluene under an argon atmosphere for 1 hour. 0.3% v/v of N, N-diisopropylethylamine (DIPEA) was employed to catalyze the hydrolysis reaction in the absence of water molecules. Finally, a curing step was performed at 110 °C for 1 hour. APTES-modified surfaces were reacted with 20 mM PDITC in a solution of 10% anhydrous pyridine in N, N-dimethylformamide (DMF) for 1 hour in darkness. Activated surfaces where either incubated with 1 μM of SH-DNA probe for 1 hour or *in-situ* biofunctionalized by flowing a solution containing a mix of SH-DNA probe and MCH at a molar ratio of 20:1 in 1 μM total concentration at a constant flow rate of 5 μL/min in 0.1 M Na_2_CO_3_ buffer at pH 9.5. The reaction was amine catalyzed by addition of 1 equivalent of triethylamine (NEt_3_) to accelerate the reaction^23^. After the incubation time, surfaces were carefully rinsed with water and dried under gentle nitrogen flux.

### Surface characterization

#### Contact angle

The contact angle measurements were performed using an Easy drop standard apparatus (Krüss GmbH, Hamburg –Germany-). Drops of Milli-Q water were placed on the Si3N4 surface with a volume of 5 μl. Each result corresponds to the average of 3 different measurements done along the Si_3_N_4_ surface. ***X-ray Photoelectron Spectroscopy (XPS) measurements***: XPS measurements were carried out with a Phoibos 150 analyzer (SPECS GmbH, Berlin, (Germany)) in ultra-high vacuum conditions (base pressure 1 × 10–10 mbar) and a monochromatic Kalpha x-ray source (81486.7 eV at a take-off angle of 0 and 54°). Data generated was analyzed by CasaXPS software. ***Fluorescence***: Biofunctionalized-Si_3_N_4_ surfaces were incubated with a solution containing 1 μM of 3′-Cyanine 3 (Cy3) modified DNA sequences complementary to the immobilized probe for 1 hour in 5× SSC buffer (pH 7). Additionally, treated Si_3_N_4_ chips interrupted at different steps along the biofunctionalization process were also incubated with the fluorescent target as control samples. Fluorescence was evaluated with a Zeiss Axio Observer Z1m optical microscope. Images of the same experiment were taken with the same parameters of camera exposition for a reliable comparison.

### Detection conditions

Isoform detection was performed by the injection of 250 μL of the samples over the surface of the BiMW biosensor at a 15 μL/min rate, with a subsequent hybridization with their complementary DNA probes immobilized on the sensor surface. Samples were dissolved in 3xSSC (0.45 M in NaCl, 0.045 M in sodium citrate) with a 45% Formamide. Finally, Isoform-probe interactions were disrupted by using a 50% formamide in aqueous solution. (WARNING: Formamide is a known carcinogen. Carry out all steps involving pure formamide manipulation in a fume hood) Calibration curves were obtained for each DNA probe by obtaining triplicates measurement of different target dilutions from standards of known concentration. The mean and the standard deviation (SD) of each concentration were plotted versus the target concentration and fitted to a dose-response curve.

### Data analysis

The data were collected using Origin 8.0 software (OriginLab, Northampton, MA). The experimental detection limit (LOD) was defined as the target concentration giving a ΔΦ (rad) in the hybridization signal at least three times higher than that of the standard deviation of the DNA/RNA control signal.

## Additional Information

**How to cite this article**: Huertas, C. S. *et al*. Analysis of alternative splicing events for cancer diagnosis using a multiplexing nanophotonic biosensor. *Sci. Rep.* **7**, 41368; doi: 10.1038/srep41368 (2017).

**Publisher's note:** Springer Nature remains neutral with regard to jurisdictional claims in published maps and institutional affiliations.

## Supplementary Material

Supplementary Information

## Figures and Tables

**Figure 1:**
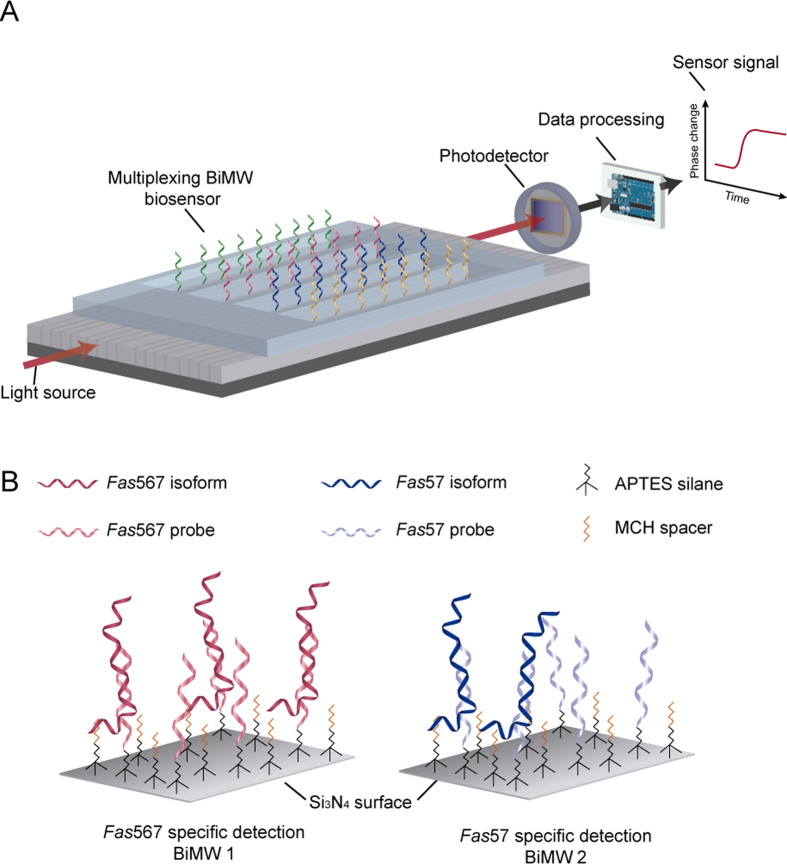
BiMW biosensor. (**A**) Scheme of the multiplexed BiMW biosensor chip with the different components for signal processing. (**B**) Scheme of the methodology followed for the direct and label-free detection of *Fas* gene alternative splicing isoforms at the biosensor surface.

**Table 1:** List of sequences for probes and DNA synthetic targets employed in this work.

Name	Sequence
*Fas*57 probe	5′-(SH)-PolyT15-CTTTCTCTTCACTTCCTCTTTG-3′
*Fas*56 probe	5′-(SH)-PolyT15-AGATCTGGATCCTTCCTCTTTG-3′
*BCL*-XL probe	5′-(SH)-PolyT15-AGTATCCCAGCCGCCGTTC 3′
*Fas*57 isoform	5′ATGTGAACATGGAATCATCAAGGAATGCACACTCACCAGCAACACCAAGTGCAAAGAGGAAGTGAAGAGAAAGGAAGTACAGAAAACATGCAGAAAGCACAGAAAGGAAAACCAAGGTTCTCATGAATCTCCAACTTTAAATCCT 3′
*Fas*567 isoform	5′ATGTGAACATGGAATCATCAAGGAATGCACACTCACCAGCAACACCAAGTGCAAAGAGGAAGGATCCAGATCTAACTTGGGGTGGCTTTGTCTTCTTCTTTTGCCAATTCCACTAATTGTTTGGGTGAAGAGAAAGGAAGTACAGAAAACATGCAGAAAGCACAGAAAGGAAAACCAAGGTTCTCATGAATCTCCAACTTTAAATCCT 3′
Cy3*-Fas*57	5′ATGTGAACATGGAATCATCAAGGAATGCACACTCACCAGCAACACCAAGTGCAAAGAGGAAGTGAAGAGAAAGGAAGTACAGAAAACATGCAGAAAGCACAGAAAGGAAAACCAAGGTTCTCATGAATCTCCAACTTTAAATCCT-**Cyanine 3**-3′

**Table 2:** Contact angle values for Untreated, APTES-mofidied, PDITC-modified and DNA functionalized Si_3_N_4_ surfaces.

Sample	Contact Angle (°)
*1. Untreated Si*_*3*_*N*_*4*_ *chip*	35.1 ± 1.19
*2. APTES-modification*	67.1 ± 0.11
*3. PDITC-modification*	59.6 ± 0.12
*4. DNA functionalization*	51.7 ± 0.82
