# Supplementary material for: Analysis of alternative splicing events for cancer diagnosis using a multiplexing nanophotonic biosensor
Source: Sci Rep. 2017 Jan 25;7:41368. doi: 10.1038/srep41368 (PMC5264646; doi:10.1038/srep41368)
Supplement: Supplementary Information [file srep41368-s1.pdf]

Supplementary Material for

Analysis of alternative splicing events for cancer diagnosis using a multiplexing nanophotonic biosensor

César S. Huertas,[1] Santos Domínguez-Zotes,[1] and Laura M. Lechuga[1]*

[1]Nanobiosensors and Bioanalytical Applications Group, Catalan Institute of Nanoscience and Nanotechnology (ICN2), CSIC, The Barcelona Institute of Science and Technology, and CIBER-BBN, Campus UAB, Bellaterra, 08193 Barcelona, Spain

* corresponding author:laura.lechuga@icn2.cat

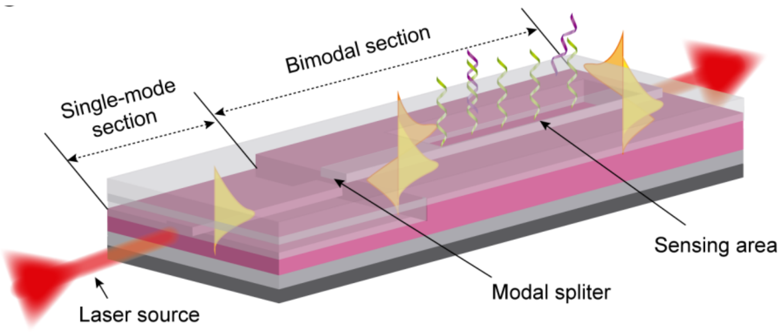

Fig. S1. Scheme of the working principle of a Bimodal Waveguide interferometer. Light is injected in the single-mode input waveguide and after a modal splitter two modes are excited and propagate until the device output.

Reagents and buffer solutions. Solvents used for sensor chips' cleaning were purchased to Panreac Applichem (Spain): Acetone 99.5%, Ethanol 99% and Methanol 99%. Main salts and chemical reagents for sensor cleaning, buffer preparation and biofunctionalization were acquired from Sigma-Aldrich (Germany): Sodium Dodecyl Sulfate (SDS), Hydrochloric Acid (HCl), anhydrous Toluene 99.8%, Sodium Phosphate monobasic (NaH_2PO_4) 99%, Sodium Phosphate dibasic (Na_2HPO_4) ≥99%, Sodium Chloride (NaCl) ≥99.5%, Sodium Citrate dihydrate (SSC) ≥99%, Sodium carbonate (Na_2CO_3) ≥99, N,N-dimethylformamide anhydrous ≥99.8%, (DMF), triethylamine ≥99% (NEt_3), Pyridine anhydrous 99.8%, crosslinking molecule p-Phenylene diisothiocyanate 98% (PDITC), 3-Aminopropyltriethoxy silane ≥98%, (APTES), *N,N*-Diisopropylethylamine (DIPEA), Mercapto-1-hexanol (MCH) 97%, and Formamide ≥99.5% (FA).

Several buffers and solvents have been prepared either for functionalization or target analysis: 20xSSC (3 M NaCl, 0.3 M sodium citrate –pH 7-), Na_2CO_3 buffer ($NaHCO_3$ 0.1 M, Na_2CO_3 0.1 M, EDTA 1mM, pH 9.2), Buffer solutions were prepared by using milliQ H_2O incubated O/N with 2% DEPC and autoclaved at 121ºC during 1 hour. All solid materials were autoclaved at 121ºC/20 min for plastic and 134ºC/10 min for glass. Biosensor microfluidics were cleaned by sequentially flowing SDS 0.5 M, HCl 0.1 M, Ethanol 95%, NaOH 0.1 M and milliQ H_2O-DEPC water.
